# Relationship between moral sensitivity and the quality of nursing care for the elderly with Covid-19 in Iranian hospitals

**DOI:** 10.1186/s12913-022-08258-x

**Published:** 2022-06-30

**Authors:** Shima Nazari, Sarieh Poortaghi, Farshad Sharifi, Shaghayegh Gorzin, Pouya Farokhnezhad Afshar

**Affiliations:** 1 School of Nursing and Midwifery, Tehran University of Medical Sciences, Tehran, Iran; 2 Elderly Health Research Center, Endocrinology and Metabolism Population Science Institute, Tehran University of Medical Science, Tehran, Iran; 3 Department of Gerontology, School of Behavioral Sciences and Mental Health (Tehran Institute of Psychiatry), Iran University of Medical Sciences, Shahid Mansouri Street, Niyayesh Street, Satarkhan Avenue, Tehran, 1445613111 Iran

**Keywords:** COVID-19, Moral status, Nurses, Quality of care

## Abstract

**Background:**

The quality of care has a significant impact on the condition of elderly patients. Many factors affect the quality of care, including ethical considerations. Ethical considerations, such as moral sensitivity, change in times of crisis. The present study was conducted to assess the relationship between moral sensitivity and the quality of nursing care for the elderly with Covid-19 in Iranian hospitals.

**Methods:**

This was a cross-sectional descriptive correlational study. The participants included 445 nurses that were selected by quota sampling method from hospitals admitting COVID-19 patients. The data were collected using the Moral Sensitivity Questionnaire (MSQ) and Quality Patient Care Scale (QUALPAC) as self-reports. We used the SPSS software v.16 for statistical analysis.

**Results:**

The total score of moral sensitivity and quality of care was 52.29 ± 16.44 and 2.83 ± 0.23, respectively. Moral sensitivity negatively correlates with psychological, social, and physical aspects (*P* < 0.05). Modifying autonomy, interpersonal orientation, and experiencing moral conflict predicted β = 0.10 of the psychosocial aspect of quality of care. Structural moral meaning and expressing benevolence predicted the changes in the physical dimension of quality of care (β = 0.02).

**Conclusion:**

The quality of care had a significant inverse correlation with moral sensitivity. Multiple regression analysis showed that modifying autonomy, interpersonal orientation, and experiencing moral conflict could predict the psychosocial dimensions. Structuring moral meaning could predict the physical dimension. The communication aspects were not related to any of the dimensions of moral sensitivity.

## Background

Coronavirus disease (COVID-19) started in 2019 and soon became a global pandemic [1]. This disease affects all populations, especially vulnerable groups like the elderly [2]. Studies have shown that older age groups are at a higher risk for infection due to comorbid conditions like diabetes, hypertension, cardiovascular disease, and cerebrovascular disease [3–6]. COVID-19 pandemics can be a challenge for health care providers, especially nurses who are at the forefront of care [7]. It has been shown that mortality increases with low quality of care [8].

The World Health Organization (WHO) defines the quality of care as “the degree to which care services for patients increase the likelihood of optimal health outcomes” [9]. The quality of care may decline in the crises such as the COVID-19 pandemic [10]. Poor quality of care can lead to patient dissatisfaction, prolonged stay, and failure to achieve desired clinical outcomes [11]. About 15% of deaths in low- and middle-income countries are due to poor quality care, but in high-income countries, less than 10% of patients are harmed while receiving hospital care [12]. One of the reasons for the reduced quality of care in pandemics is the high rate of transmission, which underlines the speed of healthcare provision. For example, Fazaeli et al. found a marked reduction in the quality of care compared to before the COVID-19 pandemic [10]. The nurses are usually under a great deal of physical and mental stress due to the high workload and continuous contact with vulnerable people in different stages of pandemic response. Studies have shown that elderly caregivers face several moral challenges in their everyday activities [13]. One of the factors that may have a pivotal role in their quality of care is moral sensitivity [14]. Moral sensitivity is a fundamental concept in the field of ethics that increases the nurses’ attention to ethical considerations in the hospital and also improves their ability to identify ethical problems and make ethical decisions [15].

Moral sensitivity is defined as awareness and attention to the existing contradictory moral values in a situation and individual self-awareness of the role and duty in that specific situation [16]. In 2020, Borges et al. studied moral injury among healthcare providers during the COVID-19 pandemic and found that healthcare providers were experiencing potential moral injuries (e.g. perception of an error and an inability to forgive oneself) that could affect their moral values [17].

Moral sensitivity is an element in identifying moral situations, reaching the stage of making sound moral decisions, and providing moral services to patients. However, it is not clear whether nurses can possess moral sensitivity during the COVID-19 pandemic; moreover, its relationship with quality of care is not well understood. Therefore, the present study was conducted to assess the relationship between moral sensitivity and the quality of nursing care for the elderly with Covid-19 in Iranian hospitals.

## Materials and methods

This was a cross-sectional descriptive correlational study (April to October 2021). The study population comprised nurses in Covid-19 wards of hospitals affiliated with Mazandaran University of Medical Sciences.

### Participants

The sample size was calculated using the formula N = [(Z_α_ + Z_β_)/C] ^2^ + 3, where C = 0.5 * Ln [(1 + r)/ (1-r)]. The r (correlation coefficient) was 0.14 according to a previous study (β = 0.2, α = 0.05) [18]. The sample size was 398 nurses. We considered 10% more sampling for the possibility of incomplete completion of questionnaires by participants (*n* = 445). The participants were selected using quota sampling in hospitals of Mazandaran University of Medical Sciences as central hospitals admitting COVID-19 patients, including Razi Hospital, Qaem Shahr (120 nurses), Imam Khomeini Hospital, Sari (107 nurses), Imam Khomeini Hospital, Amol (108 nurses), and Ibn Sina Hospital, Sari (110 nurses).

### Data collection

Data were collected using a demographic questionnaire (age, sex, marital status, degree, and work experience), the Moral Sensitivity Questionnaire (MSQ), and the Quality Patient Care Scale (QUALPAC) in a self-report manner. We explained the objectives of the study to all participants and informed consent was obtained from them.

The Moral Sensitivity Questionnaire (MSQ) was developed by Lutzen et al. (1994). It first contained 30 questions, which was reduced to 25 in subsequent modifications. It evaluates the nurses’ moral decision-making while providing clinical care in six dimensions, including “modifying autonomy” (items 10, 12, 13), “interpersonal orientation” (items 1, 2, 3, 4, 17), structuring moral meaning (items 16, 24), “experiencing moral conflict” (items 9, 11, 15), reliance on medical authority (items 6, 8, 14, 18, 20), and “expressing benevolence” (items 5, 7, 19, 21, 22, 23, 25) [19]. The items are scored on a 5-point Likert scale from completely agree (4) to completely disagree (0). The total score of the questionnaire ranges between 0 and 100. A score in the range of 0–50, 50–75, and 75–100 indicates low, moderate, and high moral sensitivity, respectively. The reliability and validity of the MSQ were previously confirmed in Iran (Cronbach’s alpha = 0.83) [20]. A Cronbach’s alpha was 0.83 in the present study.

The Quality Patient Care Scale (QUALPAC) is a 68-item scale used to evaluate the social (items 1–32), physical (items 33–55), and communicational aspects (items 56–68) of nursing care. The items are scored on a 4-point Likert scale from always (4) to never (1). The total score of the QUALPAC ranges between 68 and 272 (range for psychosocial aspect: 32–128, physical aspect: 23–92, communicational aspect: 13–52). The total score of the QUALPAC is divided by the total number of questions and the result is used to categorize the quality of care as undesirable (score of 0–1.89), relatively desirable (1.90–2.63), and desirable (2.64–4). In the original version, Content Validity Index (CVI) and Content Validity Ratio (CVR) were used to assess the content validity quantitatively (CVR = 0.81, CVI = 0.83); moreover, the scale showed excellent test-retest reliability (ICC = 0.966, *r* = 0.875) [21]. The validity and reliability of the QUALPAC were evaluated in Iran, and a Cronbach’s alpha of 0.80 was obtained [22]. In this study, Cronbach’s alpha was 0.89.

### Data analysis

Descriptive data are presented as frequency and frequency percentage. The relationship between quality of nursing care and moral sensitivity in nurses was evaluated using Pearson’s correlation coefficient and multiple regression analysis with the Enter method. The SPSS software version 16 was used for data analysis (α ≤ 0.05).

### Ethical consideration

This study has been approved by the research ethics committee of the Tehran University of Medical Sciences (Ref: IR.TUMS.FNM.REC.1400.005). We explained the objectives of the study to the participants and written informed consent was obtained from them.

## Results

The response rate was 100%. The mean age of the participants was 39.41 ± 9.61 years (range: 23–57 years). The mean age of women was 38.71 ± 9.88 and the mean age of men was 40.10 ± 9.31 (*P* = 0.12). The mean work experience of the nurses was 2.28 ± 1.08 years (range: 1–4 years). The normality test was 0.27 and 0.31 for moral sensitivity and quality of care (Kolmogorov-Smirnov test). The total score of moral sensitivity and quality of care was 52.29 ± 16.44 and 2.83 ± 0.23, respectively. According to the results, 55.5% of the nurses caring for the elderly patients with COVID-19 had low, 25.6% had moderate, and 19.9% had high levels of moral sensitivity (More information in Table 1).

**Table 1:** Mean and standard deviation of moral sensitivity and quality patient care life

	F (%)	Moral Sensitivity	Quality Patient Care
		Mean ± SD	Mean ± SD
Age	39.41 ± 9.61	–	–
*r*		0.15	0.04
*P*		0.28	0.36
Sex			
Male	220 (49.4)	51.79 ± 16.13	2.81 ± 0.23
Female	225 (50.6)	52.78 ± 16.75	2.85 ± 0.22
*P*		0.53	0.06
Education			
Associates’ degree	96 (21.6)	52.40 ± 17.18	2.83 ± 0.20
BSc. degree	162 (36.4)	52.77 ± 16.42	2.81 ± 0.23
MSc. degree	106 (23.8)	51.05 ± 15.65	2.80 ± 0.24
PhD degree	81 (18.2)	52.81 ± 16.73	2.83 ± 0.22
*P*		0.84	0.06
Marital status			
Married	217 (48.8)	51.22 ± 16.03	2.80 ± 0.23
Single	140 (31.5)	52.65 ± 17.11	2.82 ± 0.22
Divorced	68 (15.3)	52.89 ± 16.69	2.90 ± 0.17
Widow/Widower	20 (4.5)	59.35 ± 14.14	2.90 ± 0.23
*P*		0.19	0.07
work experience			
< 2 yrs.	144 (32.4)	49.65 ± 14.51	2.82 ± 0.23
2–3 yrs.	105 (23.6)	52.99 ± 16.51	2.86 ± 0.24
3–4 yrs.	124 (27.9)	53.78 ± 17.65	2.80 ± 0.21
> 4 yrs.	72 (16.2)	54.10 ± 17.45	2.83 ± 0.22
*P*		0.12	0.25

A significant inverse correlation was found between the total scores of moral sensitivity and quality of care (*P* = 0.001, *r* = −0.16). Table 2 presents the correlation of different dimensions between moral sensitivity and quality of care aspects.

**Table 2:** Correlation of moral sensitivity and quality of patient care

			Quality Patient Care		
			Psychosocial aspects	Physical aspects	Communicational aspects
		Mean ± SD	89.01 ± 12.63	66.38 ± 6.78	38.27 ± 5.34
**Moral Sensitivity**	Modifying autonomy	6.45 ± 2.82	− 0.243^**^	− 0.099^*^	0.014
	Interpersonal orientation	13.48 ± 3.36	− 0.282^**^	−0.088	0.030
	Reliance on a medical authority	2.86 ± 2.16	−0.237^**^	−0.098^*^	0.038
	Experiencing moral conflict	6.15 ± 2.51	−0.187^**^	−0.069	0.050
	Structuring moral meaning	10.95 ± 2.16	−0.211^**^	−0.124^**^	0.057
	Expressing benevolence	12.37 ± 4.72	−0.230^**^	−0.060	0.024

^*^P < 0.05

^**^P < 0.01

According to multiple regression analysis, the dimensions of “modifying autonomy”, “interpersonal orientation”, and “experiencing moral conflict” explained 0.10 of the total changes in the psychosocial aspect of care; the dimensions of “structural moral meaning” and “expressing benevolence” explained 0.02 of the total changes in the physical aspect of care. None of the dimensions of moral sensitivity could explain the Communicational aspects. (Table 3). In total, the moral sensitivity explained 0.06 of the changes in the quality of care (*P* < 0.001).

**Table 3:** Multiple regression analysis of quality of patient care dimensions and moral sensitivity

		Quality Patient Care								
		Psychosocial aspects			Physical aspects			Communicational aspects		
		B	β	*P*	B	β	*P*	B	β	*P*
**Moral Sensitivity**	Modifying autonomy	−1.87	−0.42	0.004	−0.55	− 0.23	0.13	− 0.59	− 0.31	0.06
	Interpersonal orientation	−2.28	− 0.61	< 0.001	− 0.07	− 0.03	0.84	− 0.001	− 0.001	0.99
	Reliance on a medical authority	0.15	0.02	0.87	−0.67	− 0.21	0.19	0.15	0.06	0.71
	Experiencing moral conflict	2.04	0.40	0.006	0.74	0.27	0.07	0.65	0.30	0.06
	Structuring moral meaning	.36	0.06	0.46	−0.60	−0.19	0.02	0.277	0.11	0.20
	Expressing benevolence	0.76	0.28	0.06	0.40	0.28	0.04	−0.13	−0.11	0.45
**Constant**		105.59			69.81			36.24		
**F**		9.34			2.63			1.25		
**R**^**2**^		0.11			.035			0.02		
**Adjusted R Square**		0.10			0.02			0.003		

## Discussion

The objective of this study was to determine the relationship between moral sensitivity and the quality of nursing care for the elderly with Covid-19 in Iranian hospitals. The results showed that the psychosocial aspect of care had a significant inverse correlation with all dimensions of moral sensitivity and the physical aspect of care had a significant inverse correlation with “modifying autonomy”, “reliance on medical authority”, and “structuring moral meaning”. The communicational aspect of care had no significant correlation with dimensions of moral sensitivity. Moreover, multiple regression analysis showed that “interpersonal orientation”, and “experiencing moral conflict” could predict the psychosocial aspect of care, “structuring moral meaning” and “expressing benevolence” could predict the physical aspect of care.

Moral sensitivity leads to increased nurses’ attention to ethical considerations in the quality of care [15]. In 2019, Amiri et al. found an indirect relationship between the dimensions of moral sensitivity and the quality of nursing care [13]. The nursing framework includes a commitment to care and meeting the physical and psychosocial needs of patients [23]. Moral sensitivity is a link between this commitment and the quality of care. The quality of care may decline during critical situations [10]. The guidelines cannot improve the quality of care, and there is a need for moral sensitivity to bond them.

COVID-19 affects moral issues more in the elderly [24] due to the presence of discrimination and negative attitudes about aging as well as a worse prognosis of the disease in this age group. The nurses’ attitude toward the elderly is contradictory and a negative attitude may affect all aspects of care [25]. Insufficient knowledge, experience, and equipment in older patient care can make it difficult for nurses [26]. Also, ageism in some caregivers can lead to inadequate care provision [27]. Moral challenges (e.g. decision-making in critical situations) may decrease the quality of care [28]. A possible reason for an indirect correlation between the quality of care and moral sensitivity could be that the nurses are forced to make decisions and shoulder duties that cause contradictions between personal values and professional values and codes during the COVID-19 pandemic. The present study found a moderate level of moral sensitivity in nurses caring for elderly COVID-19 patients; in another study, the same results were obtained [23]. While another study reported a high range of moral sensitivity in critical care nursing professionals [29]. This difference can be due to differences in care units (critical care unit vs. general ward). It seems that moral sensitivity is higher in critical care units than in other ward, despite the high workload. Also the study time is related to two different time periods (before and during the COVID-19 pandemic).

Nursing staff shortage is a great problem in Iran [30, 31]. We believe that a moderate level of moral sensitivity is acceptable since the quality of care was desirable despite the high workload of the nurses during the pandemic, which could be due to clinical governance and meticulous monitoring of the nursing activities in Iran that were maintained during the COVID-19 pandemic as well. Of course, the conditions and context affect its level.

This study found a significant correlation between work experience and moral sensitivity, which was consistent with a previous study [32] and may indicate that the nursing profession is associated with learning and knowledge expansion resulting in increased moral sensitivity. The results suggest that the formulation of clear guidelines regarding moral sensitivity can guarantee the quality of care. One study reported that “following the rules” and “reliance on medical authority” received the highest scores (16), which confirms the effect of guidelines on the quality of care. Nonetheless, several studies have shown the complexity of factors affecting the quality of care [13, 18, 24]; therefore, it is necessary to identify the barriers and facilitators.

## Conclusion

The present study found a moderate level of moral sensitivity and a desirable quality of nursing care in nurses caring for elderly patients with COVID-19. Quality of care had a significant inverse correlation with moral sensitivity. Multiple regression analysis showed that “modifying autonomy”, “interpersonal orientation”, and “experiencing moral conflict” could predict the psychosocial aspect of care, “structuring moral meaning” and “expressing benevolence” could predict the physical aspect of care. It is necessary to provide solutions at the organizational level (e.g. increase the number of nurses and equipment) and individual level (increase moral awareness and knowledge).

### Limitations and strengths

We tried to create diversity by sampling from different hospitals. One of the limitations of the present study was that the patients’ views on the quality of care were not assessed due to the special conditions of patients and hospitals during the COVID-19 pandemic.

## Data Availability

The datasets generated and/or analysed during the current study are not publicly available due to privacy and ethical concerns but are available from the corresponding author on reasonable request.

## Abbreviations

COVID-19: Coronavirus Disease
MSQ: Moral Sensitivity Questionnaire
QUALPAC: Quality Patient Care Scale
WHO: World Health Organization
CVI: Content Validity Index
CVR: Content Validity Ratio
